# Neutrophil extracellular trap formation during surgical procedures: a pilot study

**DOI:** 10.1038/s41598-023-42565-5

**Published:** 2023-09-14

**Authors:** Melody Ying-Yu Huang, Christoph Lippuner, Marcel Schiff, Malte Book, Frank Stueber

**Affiliations:** 1 Department of Anaesthesiology and Pain Medicine, Inselspital, Bern University Hospital, University of Bern, Bern, Switzerland; 2 Department for BioMedical Research, University of Bern, Bern, Switzerland; 3 Department of Health Sciences and Technology, Swiss Federal Institute of Technology (ETH) Zürich, Zürich, Switzerland; 4 Present Address: Universitätsklinikum Freiburg, Freiburg, Germany; 5 Present Address: Universitätsklinik für Anästhesiologie/Intensiv-/Notfallmedizin/Schmerztherapie, Oldenburg, Germany

**Keywords:** Immunology, Cardiology

## Abstract

Neutrophils can release neutrophil extracellular traps (NETs) containing DNA fibres and antimicrobial peptides to immobilize invading pathogens. NET formation (NETosis) plays a vital role in inflammation and immune responses. In this study we investigated the impact of surgical trauma on NETosis of neutrophils. Nine patients undergoing “Transcatheter/percutaneous aortic valve implantation” (TAVI/PAVI, mild surgical trauma), and ten undergoing “Aortocoronary bypass” (ACB, severe surgical trauma) were included in our pilot study. Peripheral blood was collected before, end of, and after surgery (24 h and 48 h). Neutrophilic granulocytes were isolated and stimulated in vitro with Phorbol-12-myristate-13-acetate (PMA). NETosis rate was examined by microscopy. In addition, HLA-DR surface expression on circulating monocytes was analysed by flow-cytometry as a prognostic marker of the immune status. Both surgical procedures led to significant down regulation of monocytic HLA-DR surface expression, albeit more pronounced in ACB patients, and there was a similar trend in NETosis regulation over the surgical 24H course. Upon PMA stimulation, no significant difference in NETosis was observed over time in TAVI/PAVI group; however, a decreasing NETosis trend with a significant drop upon ACB surgery was evident. The reduced PMA-induced NETosis in ACB group suggests that the inducibility of neutrophils to form NETs following severe surgical trauma may be compromised. Moreover, the decreased monocytic HLA-DR expression suggests a post-operative immunosuppressed status in all patients, with a bigger impact by ACB, which might be attributed to the extracorporeal circulation or tissue damage occurring during surgery.

## Introduction

Neutrophils comprise the largest subgroup of leukocytes and are key players in the initiation of innate immune responses, providing the first line of host cellular defence against infections^1^. Neutrophil Extracellular Trap (NET) formation, NETosis, is a relatively newly identified immune function of neutrophils. Upon activation, neutrophils release decondensed chromatin and various granule proteins that together form an extracellular fibril matrix—NET. NETs stimulate immune functions by (1) binding to extracellular pathogens to prevent further spreading and (2) maintaining a high concentration of antimicrobial components locally^2,3^.

Clinically, NETs have been associated with different types of infections and are involved in cardiovascular and inflammatory diseases^4–7^. Recently, NETs were thought to be responsible for uncontrolled tissue damage and thrombotic responses in severe pulmonary complications of coronavirus disease-19 (COVID-19)^8–11^. Further, there are indications that overproduction of NETs may have deleterious effects on sepsis-induced organ injury and mortality^12–14^. Besides infections, emerging evidence has suggested that NETs might also be involved in other non-infectious diseases such as tumour-associated thrombosis and deep vein thrombosis, cancer progression and metastasis, autoimmune diseases, diabetes and cardio-metabolic disorders, and atherosclerosis^15–23^.

Surgical procedures can result in a variety of metabolic and endocrine responses, including an immunosuppressive effect on patients during the immediate post-operative period, which may further be implicated in the development of post-operative septic complications and tumour metastasis formation^24–27^. Although NETosis has been shown to be involved in various immune processes, including the pathogenesis of sepsis^28–30^, until now its formation during an immunosuppressed state such as perioperative surgical trauma remains largely unclear. It is conceivable that NETosis may be compromised during a state of immunodeficiency. Thus, the aim of this study was to investigate whether perioperative immunosuppression as described above could be reflected in the NETosis rate in patients who underwent mild or severe surgical trauma.

## Methods

### Study participants and surgery

Study participants included nine “transcatheter/percutaneous aortic valve implantation” patients (TAVI/PAVI) and ten “aortocoronary bypass” patients (ACB). TAVI/PAV is a minimally invasive procedure which is considered mild surgical trauma, whereas ACB is open-heart surgery with a heart–lung machine and is considered severe surgical trauma. The 19 patients were included in our pilot study, which was approved by the local ethical committee Kantonale Ethikkommission Bern (KEK Berne, 041/09) and all research was performed in accordance with relevant guidelines/regulations. Each patient was scheduled for either surgical or trans-catheter treatment at our institution. A detailed description of both treatments can be found in Erdoes et al.^31^. All patients signed written informed consent.

Inclusion criteria were written informed consent, age > 18 years, Caucasian, bypass surgery using minimal extracorporeal circulation or aortic valve replacement using TAVI/PAVI under sedation. Exclusion criteria were no written informed consent, steroid medication, known immune dysfunction or suppression, and acute infection.

### Blood collection

9 ml of EDTA blood was collected at induction of anaesthesia (0 h), by the end of the surgery (closing of the sternum, or end of the TAVI/PAVI), 24 h and 48 h post induction.

### Neutrophil purification

Neutrophils were purified from the fresh EDTA blood as described by Brinkmann and colleagues^32^. Briefly, 7 ml of EDTA peripheral whole blood was layered onto 6 ml Histopaque® 1.119 g/ml (Sigma Aldrich, Switzerland) and centrifuged at 800×*g* for 20 min. Subsequently, the samples from the phase containing the granulocytes were collected and washed with Dulbecco’s phosphate buffered saline (DPBS) (Sigma Aldrich, Switzerland). Afterwards, the cells were re-suspended in 2 ml DPBS. The cell suspension was then layered onto a Percoll® (Sigma Aldrich, Switzerland) gradient (85%, 80%, 75%, 70% and 65% in DPBS) and centrifuged at 800×*g* for 20 min. The top layer, up to 65%, was removed, and the remaining layers, including most of the top 85% layer, were transferred to a 50 ml tube. After another washing step with DPBS, the cells were re-suspended in 2 ml RPMI1640 (Sigma Aldrich, Switzerland) containing 2% human serum albumin (Sigma Aldrich). After counting the living cells, 2 × 10^5^ cells (in 500 µl medium) were seeded into a 24-well plate (Techno Plastic Products AG, Switzerland) containing round glass cover slides and incubated for 30 min at 37 °C, 5% CO_2_. Afterwards, the cells were stimulated with 50 nM PMA (Sigma Aldrich, Switzerland) for 2 h at 37 °C, 5% CO_2_. Subsequently, the cells were fixed with 4% paraformaldehyde (AppliChem, Germany) for 10 min, washed twice with DPBS and stored at 4 °C for later staining.

### Neutrophil extracellular trap-staining for fluorescence microscopy

The staining of the neutrophil extracellular traps was performed as described by Brinkman and colleagues^32^. Briefly, the glass cover slides with the fixed neutrophils were removed from the 24-well plate and placed onto a drop of permeabilisation buffer containing 0.5% Triton-X100 (VWR, Switzerland)/DPBS for 1 min and immediately washed 3 times on DPBS drops. The cells were then incubated in the blocking buffer containing 3% donkey serum (Sigma Aldrich, Switzerland), 3% cold-water fish gelatin (Sigma Aldrich, Switzerland), 1% BSA (Roche Life Science, Switzerland), and 0.05% Tween-20 (AppliChem, Germany) for 20 min. Afterwards, the cells were stained with rabbit anti-neutrophil elastase (diluted 1:200, Abcam, United Kingdom) and mouse-anti-chromatin PL2-3 supernatant (diluted 1:100, kindly provided by Dr. Brinkmann, Berlin) at room temperature for 2 h, then washed 3 times by placing the cover glasses on DPBS drops. For detection, the secondary antibodies, the donkey anti-rabbit AlexaFluor®488 (diluted 1:200, Abcam, United Kingdom) and the donkey anti-mouse AlexaFluor®594 (diluted 1:600, Abcam, United Kingdom) were diluted in blocking buffer and added to the cells for 1-h staining in the dark at room temperature. Afterward the DNA was stained with Hoechst® 33,342 (Thermo Fisher Scientific, Switzerland, diluted 1:40,000 in water) and the cover slips were mounted on glass slides in Mowiol (VWR, Switzerland) containing 1,4-Diazabicyclo [2.2.2] octan (DABCO; Sigma Aldrich, Switzerland). Controls with only the secondary antibodies were included.

### Fluorescence microcopy

The slides were recorded on an Eclipse 80i microscope (Nikon, Japan) at a 20-fold magnification. Five regions were photographed from each slide. The pictures were then analyzed with ImageJ v1.51r (National Institutes of Health, USA), as described in Brinkmann and co-workers^33^. The cells were counted in the blue Hoechst channel by thresholding the pixel intensity using the Bernsen algorithm (radius 15 pixels, minimum pixel intensity 35) and counting the events with a minimum size of 20 pixels.

NETosis-positive cells (chromatin stained) were selected in the red channel by setting a threshold for pixel intensity. Cells undergoing NETosis decondensated their chromatin, making them more accessible to the antibody and thus giving a stronger fluorescent signal. For single pictures, the results are given as the percentage of NETosis rates, which were calculated by the NETosis-positive cell count divided by the total cell count.

### Quantitative HLA-DR staining

50 µl EDTA freshly drawn peripheral whole blood were stained with 20 µl Quantibrite™ Anti-HLA-DR PE (phycoerythrin) and Anti-Monocyte PerCP (peridinin chlorophyll protein)-Cy™5.5 (BD Biosciences, Europe) in the dark at room temperature for twenty-five minutes. Red blood cells were subsequently lysed with FACS lysis solution (BD Biosciences, Europe) for five to ten minutes, washed twice with DPBS (Sigma-Aldrich, Switzerland), and fixed with 400 µl 4% paraformaldehyde in PBS. One unstained control was also measured for each patient and at each time point. The fluorescence intensity of the samples was measured in duplicates on a LSR II (BD Biosciences) using the software FACSDiva v6.1.3 (BD Biosciences, Europe). 1000 monocyte events were recorded. For the absolute quantification of antibodies bound per cell, the Quantibrite™ PE fluorescence beads (BD Biosciences) were used in each flow cytometric recording. Data were analyzed using FlowJo v7.5 software (Tree Star Inc., Ashland, OR, USA) with monocytes gated by anti-monocyte PerCP-Cy5.5 positive cells. The PE channel was calibrated with the PE Beads, and the fluorescence intensity was correlated with the mean number of the PE molecules per cell. The results were recorded as the median of the calibrated PE channel fluorescence intensity of each sample. The mean and standard deviation were calculated for the duplicate samples.

### Inflammatory biomarker analysis

The quantification of the cytokines and chemokines in the plasma (EDTA) was performed using Human Focused 11-Plex Discovery Assays® (Eve Technologies, Canada). Briefly, the bead-based multiplexing technology, also known as addressable laser bead immunoassay (ALBIA), applied different combinations of fluorophore beads conjugated to specific antibodies targeting the cytokines and chemokines of interest at once. The 11-plex consisted of GM-CSF, IFN-γ, IL-1β, IL-2, IL-4, IL-6, IL-8, IL-10, IL-12, MCP-1, and TNF-α. All eleven markers were simultaneously measured in the samples in duplicates and the average values were taken as final measurement. The assay was performed by Eve Technologies, Canada.

### Statistical analysis

The analyses of the repeated measures data were performed using either two-way repeated measures (RM) ANOVA—when data were normally distributed and contained no missing values—or otherwise based on a mixed-effects model, which uses the restricted residual maximum likelihood method (REML). For post-hoc analyses, either Turkey’s within-group multiple comparison test correction or Šídák’s between-group test correction was applied. Data are presented as box plots with whiskers showing median, interquartile ranges, and minimum to maximum bars, with all data points displayed as round dots. The GraphPad Prism software version 9.5.0 (730), November 2022 (GraphPad Software, LLC, USA) was used for the statistical analysis and graphing of the results.

## Results

### Demographics

During the study period, ten patients undergoing aortocoronary bypass surgery (ACB, severe surgical trauma), nine patients undergoing transcatheter aortic valve implantation (TAVI, mild surgical trauma) and one patient undergoing percutaneous aortic valve implantation (PAVI, mild surgical trauma) were included.

The perioperative characteristics of patients’ in the two groups did not differ with regard to the American Society of Anesthesiologists (ASA) category^34^ (Table 1). As is typical, patients in the ACB group were younger than those in the TAVI/PAVI group: 65.5 years [59.0; 73.5] versus 83.0 years [79.0; 87.0] (median with interquartile range); moreover, the surgical procedure lasted longer in the ACB group than in the TAVI/PAVI group: 220.5 min [210.8; 244.8] versus 66.0 min [61.5; 81.5], respectively (Table 1).

**Table 1:** Patients and procedural characteristics.

	TAVI/PAVI (n = 9)	ACB (n = 10)	p
Age, yrs	83.0 [79.0; 87.0]	65.5 [59.0; 73.5]	< 0.0001
Sex, n (m/f)	3/6	10/0	0.0018
ASA^a^ III/ASA IV	2/7	4/6	ns
Procedural time, mins	66.0 [61.5; 81.5]	220.5 [210.8; 244.8]	< 0.0001
ECC time, mins	0	83.0 [69.5; 101.3]	< 0.0001

*ASA* indicates American Society of Anesthesiologists, *ECC* extracorporeal circulation, *ns* non-significant.

Values are numbers or median with 25%, 75% interquartile range. p < 0.05 is considered significant.

^a^Classification according to guidelines^34^.

### Monocytic HLA-DR surface expression

**Figure 1:**
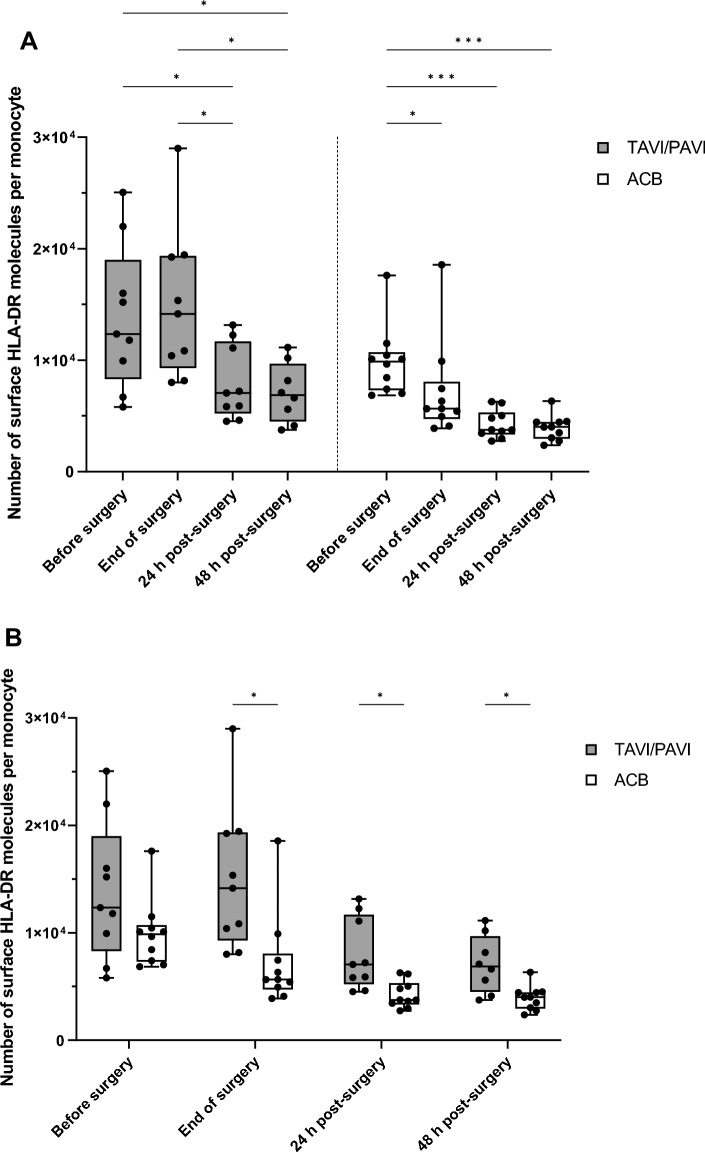
HLA-DR surface expression on circulating monocytes. Flow cytometric estimation of the HLA-DR antibodies bound per monocyte. The box and whisker plots of the dataset analysed by a mixed-effects model (REML). Filled box: TAVI/PAVI group, n = 9; unfilled box: ACB group, n = 10. The centre line denotes the median value (50th percentile), while the box contains the 25th to 75th percentiles of dataset. The whiskers mark the minimum and maximum values, and all data points are marked with dots. The four different measured time points were before surgery (0 h, during induction of anaesthesia), end of surgery (closing of sternum, or end of TAVI/PAVI), 24 h and 48 h after surgery. (**A**) Post-hoc analysis with Turkey’s multiple comparison test correction revealed significant regulations of monocytic HLA-DR expression within each surgical group (*p < 0.05, **p < 0.01, ***p < 0.001). (**B**) Post-hoc analysis with Šídák’s multiple comparison test correction revealed significant differences of monocytic HLA-DR expression between the two surgical groups by the end of and after surgery (*p < 0.05, **p < 0.01, ***p < 0.001).

In the TAVI/PAVI group, HLA-DR surface expression on monocytes first slightly increased by the end of surgery, followed by a significant drop both at 24 h and 48 h post-surgery (REML analysis with Turkey’s multiple comparisons test, *p* = 0.047 and *p* = 0.023, respectively, Fig. 1A). In contrast, in the ACB group, HLA-DR expression decreased significantly already by the end of surgery and decreased further both at 24 h and 48 h post-surgery (REML analysis with Turkey’s multiple comparisons test, end of surgery: *p* = 0.0407, 24 h post-surgery: *p* = 0.0002, 48 h post-surgery *p* = 0007, Fig. 1A). When comparing the same time points between the two groups, HLA-DR expression was the same for both groups before surgery. However, the HLA-DR levels on monocytes by the end of and after surgery were significantly reduced in ACB patients when compared to the TAVI/PAVI group (REML analysis with Šídák’s multiple comparisons test, before surgery: *p* = 0.4094, end of surgery: *p* = 0.0445, 24 h p.s.: *p* = 0.0407, 48 h p.s.: *p* = 0.0472, Fig. 1B).

### NET formation rate

**Figure 2:**
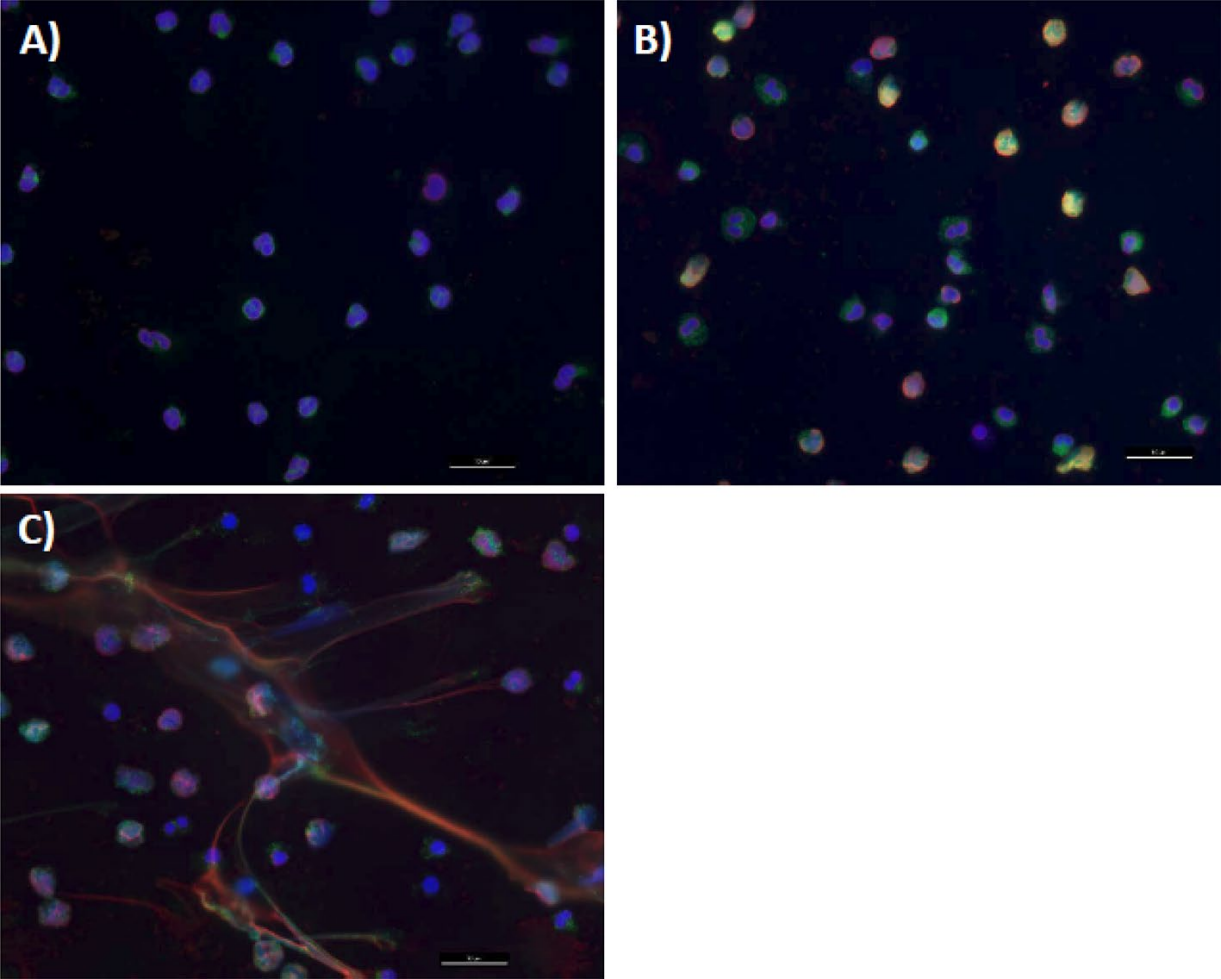
Microscopic analysis of NET formation. (**A**) Unstimulated neutrophils with intact, segmented nuclei. (**B**) Neutrophils stimulated with 50 nM PMA for 2 h showing loss of nuclear lobules and loss of nucleus integrity that led to an increase in overlapping staining of nuclei (histone H2B) and granule proteins (neutrophil elastase). (**C**) PMA-stimulated neutrophils with extensive NET formation. Photos were taken with 20X magnification, and the scale bar represents 10 µm. Fluorescent antibodies used in staining: Hoechst 33,342 for DNA (blue), rabbit anti-neutrophil elastase (green), and mouse anti-histone H2B (red).

NET formation was analysed by fluorescence microscopy (Fig. 2). Unstimulated neutrophils showed an intact nucleus and almost no staining for histones or granule (i.e. neutrophil elastase, Fig. 2A). Two-hour stimulation of neutrophils with 50 nM PMA led to morphological changes during the process of NETosis. The cells were flattened, losing their nuclear lobules and granules (i.e. enhanced neutrophil elastase staining) as well as nucleus integrity (i.e. enhanced histone staining) (Fig. 2B). The ejected neutrophil extracellular traps could be observed following a pronounced NETosis (Fig. 2C).

**Figure 3:**
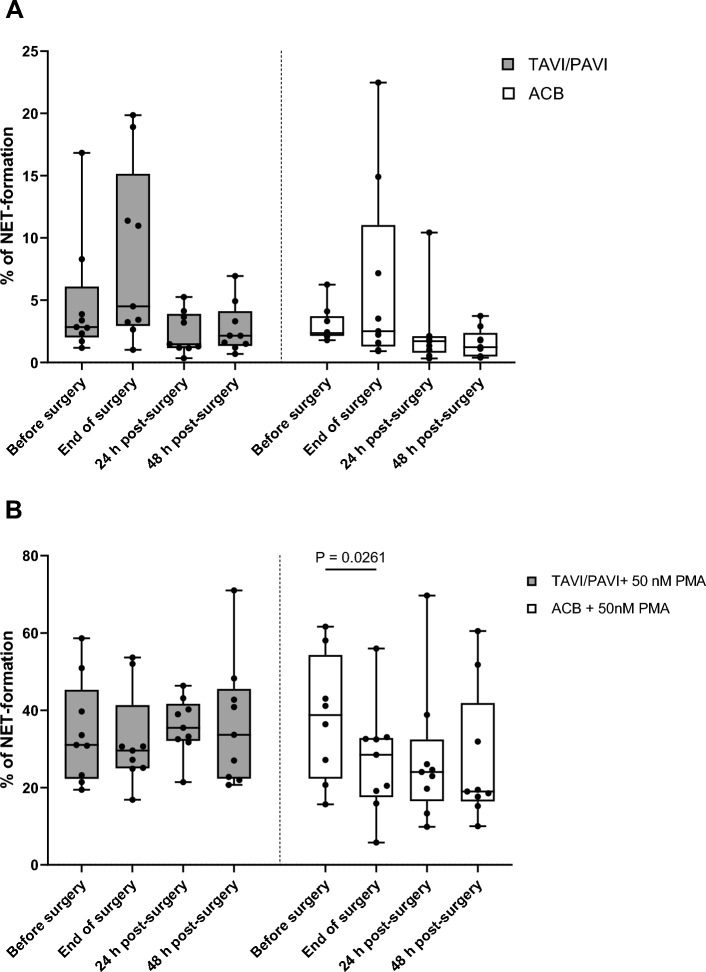
Neutrophil NETosis rate at different surgical time points with or without PMA induction. (**A**) Spontaneous NETosis rate of neutrophils without stimulation. The box and whisker plot of the dataset analysed using two-way repeated measures ANOVA, which revealed a highly significant time factor of the variance (*p* = 0.0043) and a significant individual difference (*p* = 0.018). Filled box: TAVI/PAVI group, n = 9; unfilled box: ACB group, n = 9. The centre line denotes the median value (50th percentile), while the box contains the 25th to 75th percentiles of dataset. The whiskers mark the minimum and maximum values, and all data points are marked with dots. The four different measured time points were before surgery (0 h, during induction of anaesthesia), end of surgery (closing of sternum, or end of TAVI/PAVI), 24 h and 48 h after surgery. (**B**) NETosis rate of neutrophils upon 50 nM PMA stimulation The box and whisker plot of the dataset was analysed using a mixed-effects model (REML). Filled box: TAVI/PAVI group, n = 9; unfilled box: ACB group, n = 9. The centre line denotes the median value (50th percentile), while the box contains the 25th to 75th percentiles of dataset. The whiskers mark the minimum and maximum values, and all data points are marked with dots. Post-hoc analysis with Turkey’s multiple comparison test correction revealed a significant drop (*p* = 0.0261) in neutrophil inducibility of NETosis by the end of ACB procedure.

To quantify the NETosis, we computed the NETosis rate as detailed in the “Methods” section. In both surgical groups, there was a trend toward increased spontaneous NETosis by the end of surgery, followed by a decrease post-surgery (Fig. 3A). Two-way RM ANOVA revealed a highly significant time factor (*p* = 0.0043) and significant patient variance (*p* = 0.0180). Post-hoc multiple comparisons did not identify any significant within-group differences between two time-points or between-group differences at single time points (Turkey’s and Šídák’s multiple comparison tests, respectively). Overall, the time effect had no significance on the PMA-induced NETosis, and no obvious trend was seen in the TAVI/PAVI group (REML analysis, Fig. 3B). Nonetheless, a decreasing trend of toward PMA-induced NETosis was seen in the ACB group, with a significant drop in the end the surgery (*p* = 0.0261, REML analysis with Šídák's multiple comparisons test, Fig. 3B).

**Figure 4:**
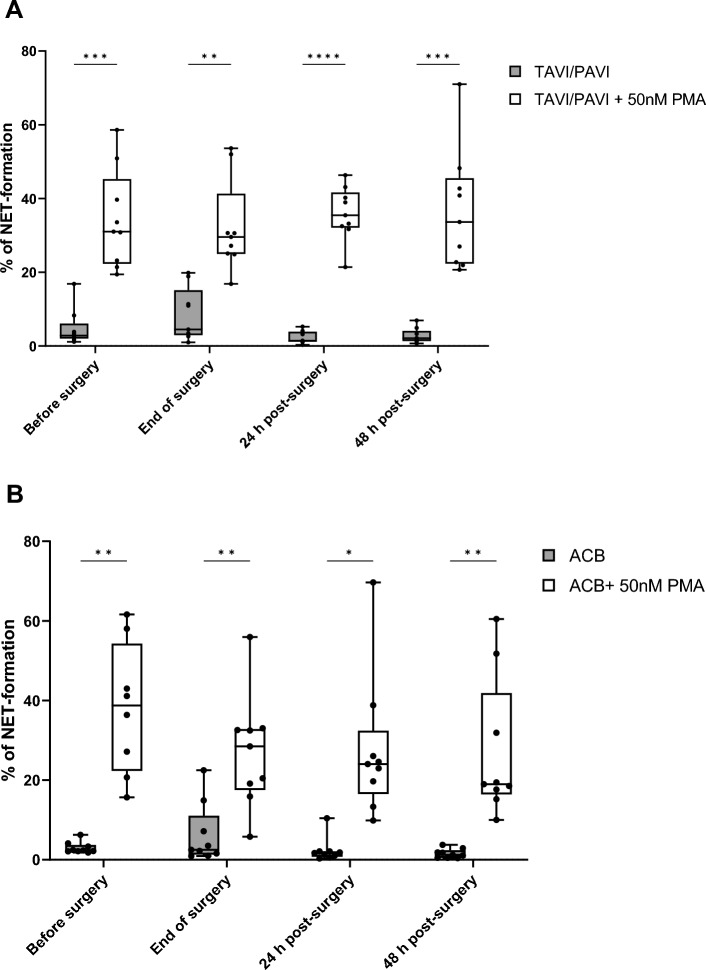
Neutrophil NETosis inducibility at different surgical time points upon PMA induction. (**A**) Neutrophil NETosis inducibility over the time course of the TAVI/PAVI surgery. The box and whisker plot of the dataset analysed by two-way repeated measures ANOVA, which revealed a highly significant source of variation of both PMA induction and individual difference (both *p* < 0.0001). Filled box: spontaneous NETosis in TAVI/PAVI patients, n = 9; unfilled box: PMA-induced NETosis in TAVI/PAVI patients, n = 9. The centre line denotes the median value (50th percentile), while the box contains the 25th to 75th percentiles of dataset. The whiskers mark the minimum and maximum values, and all data points are marked with dots. The four different measured time points were before surgery (0 h, during induction of anaesthesia), end of surgery (closing of sternum, or end of TAVI/PAVI), 24 h and 48 h after surgery. Post-hoc analysis with Šídák’s multiple comparison test correction revealed highly significant induced NETosis upon PMA stimulation across all surgical time points (**p < 0.01, ***p < 0.001). (**B**) Neutrophil NETosis inducibility over the time course of the ACB surgery. The bar figure of the dataset analysed by a mixed-effects model (REML), which revealed a highly significant type III fixed effect of PMA stimulation and significant effects of both time and time x PMA-stimulation interaction (*p* = 0.0001, 0.0409, and 0.0187, respectively) on the inducibility of neutrophils NETosis. Filled box: spontaneous NETosis in ACB patients, n = 9; unfilled box: PMA-induced NETosis in ACB patients, n = 9. The centre line denotes the median value (50th percentile), while the box contains the 25th to 75th percentiles of dataset. The whiskers mark the minimum and maximum values, and all data points are marked with dots. The four different measured time points were before surgery (0 h, during induction of anaesthesia), end of surgery (closing of sternum, or end of TAVI/PAVI), 24 h and 48 h after surgery. Post-hoc analysis with Šídák’s multiple comparison test correction revealed (highly) significant induced-NETosis upon PMA stimulation across all surgical time points (*p < 0.05, **p < 0.01).

To examine the inducibility of neutrophils to form NET structure following different surgical procedures, for both surgical groups, we further performed independent repeated measures analyses to compare neutrophils’ NETosis rates with and without PMA stimulation. In the TAVI/PAVI group, both PMA stimulation and individual differences were highly significant factors for the NETosis (both *p* < 0.0001, two-way RM ANOVA, Fig. 4A). In the ACB group, in additional to the PMA stimulation and individual variation, the time effect and time and PMA stimulation interaction were also proven to be significant factors for NETosis (REML analysis, fixed effects: *p*_(time)_ = 0.0409, *p*_(PMA stimulation)_ = 0.0001, *p*_(time x PMA stimulation)_ = 0.0187 and random effects: SD/Variance_patient_ = 10.51/110.4, SD/Variance_residual_ = 5.821/33.89, Fig. 4B).

### Correlational study

Since monocytic HLA-DR expression appeared to be a more promising indicator of immune status compared to the neutrophilic NETosis-related endpoints (i.e. spontaneous NETosis and PMA-induced NETosis), we wanted to know whether there is correlation between these two leukocytes’ immune responses to the underlying surgical trauma. Thus, we conducted a series of Spearman nonparametric correlation analyses (Supplementary Fig. S1–S3). The overall data across the surgical time indicated that monocytic HLA-DR expression was positively correlated with the spontaneous neutrophilic NETosis, and HLA-DR had no correlation with PMA-induced NETosis in the TAVI/PAVI patients but a slight positive correlation in the ACB patients. When looking at different surgical timepoints, in the ACB group, the monocytic HLA-DR expression showed no correlation with the PMA-induced NETosis before surgery but a strong negative correlation in the end of surgery, followed by a strong positive correlation 24 h post-surgery (Supplementary Fig. S2). Moreover, we also compared the same endpoint across surgical time and found that, for most endpoints, there were positive correlations between different time points- (Supplementary Fig. S3).

### Inflammatory biomarker analysis

**Figure 5:**
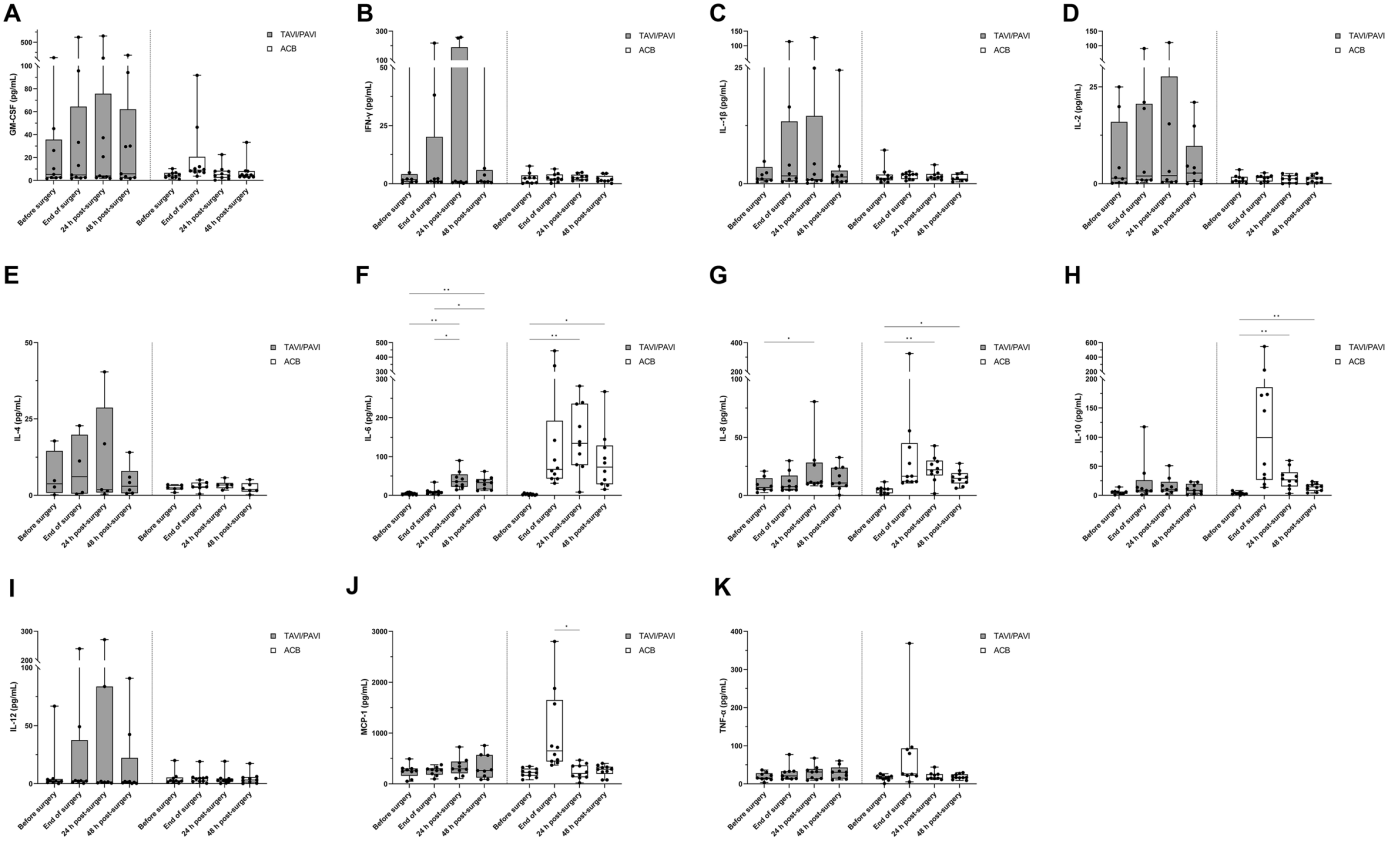
Inflammatory cytokine and chemokine analysis in the plasma. (**A-K**) Flow cytometric estimation of eleven inflammatory biomarkers in the plasma (EDTA) samples of both TAVI/PAVI and ACB patients. The box and whisker plots of the dataset analysed either by a mixed-effects model (REML; (**A**-**E**), (**G**-**I**), and (**K**)) or by two-way repeated measures ANOVA (**F** and **J**). Filled box: TAVI/PAVI group, n = 9; unfilled box: ACB group, n = 10. The centre line denotes the median value (50th percentile), while the box contains the 25th to 75th percentiles of dataset. The whiskers mark the minimum and maximum values, and all data points are marked with dots. The four different measured time points were before surgery (0 h, during induction of anaesthesia), end of surgery (closing of sternum, or end of TAVI/PAVI), 24 h and 48 h after surgery. Post-hoc analysis with Šídák’s multiple comparison test correction revealed significant regulations of IL-6, IL-8, IL-10, and MCP-1 extracellular secretion within a surgical group (*p < 0.05, **p < 0.01, ***p < 0.001).

To further examine inflammatory biomarkers following both surgical procedures, we performed a series of cytokine/chemokine analyses in the EDTA plasma samples from both patient groups. A total of eleven inflammatory biomarkers of choice were tested (see “Methods”). The results showed very distinct regulation patterns between the two surgical traumata. TAVI/PAVI seemed to trigger more GM-CSF, IFN-γ, IL-1β, IL-2, IL-4, and IL-12 production, while IL-6, IL-8, IL-10, MCP-1, and TNF-α were mostly increased following the ACB procedure (Fig. 5). Among the inflammatory markers, specially, cytokine IL-6 (two-way RM ANOVA analysis, *p*_(time)_ = 0.0037, *p*_(surgical procedure)_ = 0.0013, *p*_(time x surgical procedure)_ = 0.015), cytokine IL-10 (REML analysis, *p*_(time)_ = 0.0198, *p*_(surgical procedure)_ = 0.0238, *p*_(time x surgical procedure)_ = 0.0104), chemokine MCP-1 (two-way RM ANOVA analysis, *p*_(time)_ = 0.011, *p*_(time x surgical procedure)_ < 0.0001,* p*_(patient)_ = 0.0165), and cytokine TNF-α (REML analysis, *p*_(time x surgical procedure)_ = 0.0473) revealed a significant difference between the two surgical procedures and/or the surgical procedure and time interaction.

Last but not the least, we correlated the biomarker measurements with the monocytic HLA-DR and the NETosis endpoints. Overall, the monocytic HLA-DR expression showed a significant negative correlation with the plasma IL-6 expression in both surgical groups (Fig. S4). Spontaneous NETosis in the TAVI/PAVI group was negatively correlated with IL-6 and IL-12, while it was positively correlated with MCP-1 and TNF- α in ACB. The PMA-induced NETosis only showed significant positive correlation with GM-CSF and MCP-1 in TAVI/PAVI patients.

## Discussion

The present study demonstrates that neutrophils of patients who experienced severe or mild surgical trauma can form neutrophil extracellular traps upon PMA stimulation. Neutrophilic granulocytes have been studied for decades regarding their migration, granule release and phagocytic activity. The fact that neutrophils can eject their own DNA in a suicidal process has been described relatively recently^2,16^. The ejected DNA fibres of neutrophils are associated with several proteins that adhere to the DNA. These include more than 30 components of different neutrophil granules, such as, myeloperoxidases or neutrophil elastases, which show bactericidal activity^3^.

Neutrophil extracellular traps may contribute to the pathogenesis of different infections and other diseases^4–10,15–23,35^. Specifically, NETosis has been shown to be involved in the pathogenesis of sepsis^14,28–30^, and one report described that neutrophils of septic and burned patients showed spontaneous NETosis and were unresponsive to several stimuli such as LPS-activated platelets or pro-inflammatory TNF-α^36^. However, it remains unknown how a surgical trauma influences the rate of NETosis. Surgical trauma can lead to postoperative immune suppression^24,31,37^. In addition, different drugs used during surgical processes are known to inhibit major functions of neutrophils^38–40^. This led to the hypothesis that severe surgical trauma, such as aorta coronary bypass surgery, might result in stronger suppression of neutrophil extracellular trap formation when compared to neutrophils from patients who undergo comparatively mild surgical trauma, such as transcatheter or percutaneous aortic valve implantation.

In the study, the absolute monocytic HLA-DR surface molecules were quantified for every recruited patient at four different time points for each of the two surgical procedures. The results showed no significant difference at the time of induction (0 h) between both surgical traumas, but a significant reduction of HLA-DR expression in the patients who underwent severe surgical trauma by the end of the surgery and both 24 h and 48 h post-surgery, compared to the mild surgical group. During the mild surgery, HLA-DR expression first increased slightly and then decreased at 24 h and 48 h post-surgery. However, HLA-DR expression was already significantly downregulated by the end of the severe surgery, and the reduction became even more pronounced post-surgery. This was typical for an inflammatory reaction that is non-pathogen-induced, which indicates a compromised ability of APCs like the circulating monocytes to present foreign antigens to certain lymphocytes such as T cells^41^. These results suggest that the immune function of patients who undergo severe surgery is significantly more suppressed. In the case of the ACB patients, this could potentially be caused by the extracorporeal circulation or by tissue damage associated with the surgery.

In parallel with monitoring the immune function of monocytes, neutrophils of the patients were isolated and stimulated ex vivo with PMA (widely used for experimental induction of NETs) to evaluate their NET-forming capacity. Neutrophil extracellular traps are DNA structures ejected from the neutrophils after chromatin decondensation^2^. Non-induced neutrophils showed segmented and compacted polymorphic multi-lobed nuclei with condensed DNA (DNA staining in blue, Fig. 2A). After PMA-induction, the DNA signals became more and more distributed in a time-dependent manner (Fig. 2B,C). Due to the decondensation and ejection of DNA, the histones and other intracellular components such as neutrophil elastases were more accessible to the immunofluorescence staining, resulting in a stronger red (anti-histone H2B) and green (anti neutrophil elastase) signals (Fig. 2).

The average age of the TAVI/PAVI patients undergoing mild surgical trauma was about 18 years older than the ACB group (severe surgical trauma), which might result in a lower “baseline” NETosis rate (i.e. before surgery, 0 h) compared to the younger ACB group, since elderly people generally display reduced neutrophil functionality^42^. However, in fact, we did not observe any significant differences in spontaneous NETosis between the groups, nor did we find a different PMA-induced NETosis before surgery. Furthermore, neutrophils from both patient groups across all surgical time points could be induced with PMA to form NETs (Fig. 5A and B). Worth mentioning is the fact that we observed similar trends in increased spontaneous NETosis by the end of both surgeries, followed by the opposite trends in both groups post-surgery (Figs. 3A and 4), and the statistical analyses indeed identified (surgical) time as a significant source of variance. For the PMA-induced NETosis, on the other hand, time was no longer a significant factor, and no clear regulating trend was seen in the mild surgical group. However, a decreasing trend in PMA-induced NETosis was observed in the severe surgical group (Fig. 3B). Taken together, both mild and severe surgical traumas have a comparable impact on spontaneous NETosis of neutrophils. The ability of neutrophils to be induced to form NETs may be compromised following severe surgical trauma but not mild surgery. Both the monocytic and neutrophilic immune endpoints suggest that the severity of the surgical trauma influences the modulation of both the innate and adaptive cellular immunity, and that the capacity of neutrophils to form NET structures upon PMA induction may be reduced after a major surgical trauma. If this compromised function of neutrophils can be attributed to a higher risk of sepsis after surgery, further investigations will be required.

Certain anesthetics are known to inhibit neutrophils’ immune function^39,40^, therefore, they may potentially reduce NET formation during the surgical process. Moreover, the compromised neutrophilic NET formation following surgical trauma may be related to the immunosuppressive mechanism via adenosinergic pathway^43,44^, since NET formation depends on glycolytic ATP production for microtubule network rearrangements^45,46^. Further investigation of drug-reduced NET formation and/or mitochondrial ATP production and NET formation during immunosuppression in the immediate post-operative period will help us understand underlying NET formation regulatory mechanisms, both in physiological and critical immune conditions.

According to the correlation analyses, monocytic HLA-DR expression in the ACB patients had no correlation with PMA-induced NETosis before surgery, but a high negative correlation in the end of surgery and a high positive correlation 48 h after surgery. Moreover, over the surgical time course, single NETosis-related endpoints in the end of and/or post-surgery were mostly correlated with their before-surgery status. All these results may imply that, during severe surgical trauma, the inducibility of neutrophil NETosis may timely reflect the surgery-related changes in the immune status (e.g. HLA-DR expression), and the native individual immunity might successfully predict the post-surgery immune responses, including the critical recovery phase. To gain a deeper understanding of how NETosis’ regulation may reflect patients’ immune status during critical care, a more comprehensive and systematic study on the topic will be needed.

Certain limitations in the current study must be considered when interpreting the results. First, PMA was chosen as the sole stimulus to induce neutrophils to form NETs. Although widely used for such a purpose as described in many published studies, PMA is an artificial inducer that bypasses membrane receptors and thus related signalling molecules. Future studies may consider more natural stimuli, such as bacteria or other pathogens, which may induce a more systematic immune response in the stimulated cells. Moreover, studying a greater number of patients would not only allow more powerful statistics, but would provide an opportunity to identify other involved immune modulators upon surgical trauma such as genetic polymorphisms in individuals.

## Conclusion

Both mild and severe surgical traumas influence HLA-DR surface expression on monocytes, however with the latter to a greater extent. Spontaneous NETosis of neutrophils is timely regulated upon surgical trauma, and a similar trend of regulation is seen in both surgical procedures. The inducibility of neutrophils to form NETosis upon stimulation is not influenced during mild surgical trauma but may be compromised following severe surgical trauma.

### Supplementary Information

Supplementary Figures.

Supplementary Table S1.

Supplementary Table S2.

## Data Availability

The data presented in this study are available on reasonable request from the corresponding author.
