# Supplementary material for: Neutrophil extracellular trap formation during surgical procedures: a pilot study
Source: Sci Rep. 2023 Sep 14;13:15217. doi: 10.1038/s41598-023-42565-5 (PMC10502064; doi:10.1038/s41598-023-42565-5)
Supplement: Supplementary file 1 — Supplementary Figures. [file 41598_2023_42565_MOESM1_ESM.pdf]

Supplementary figures

Supplemental figure 1

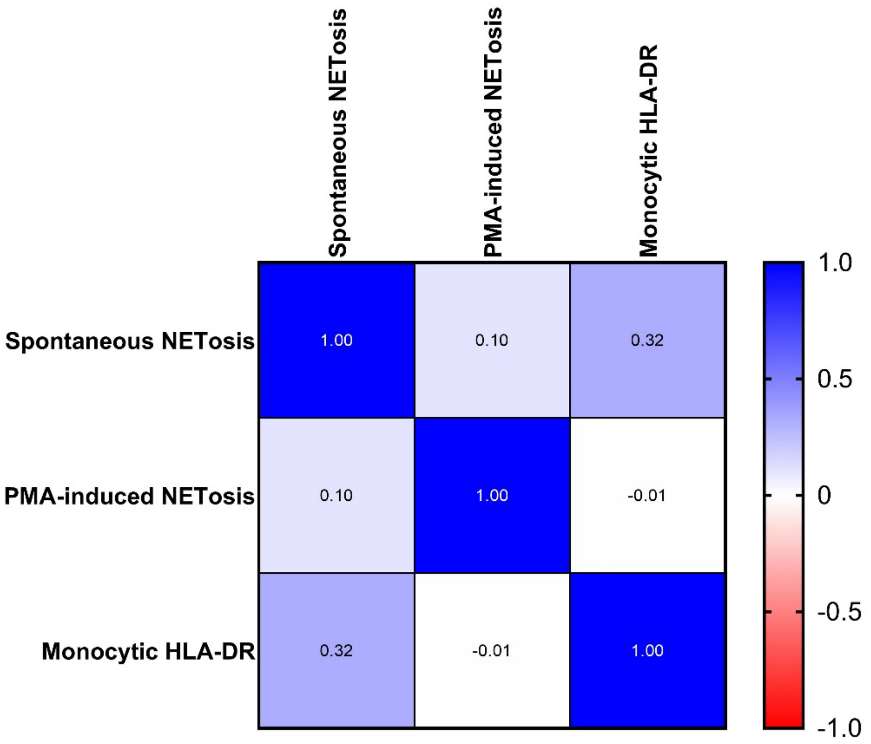

Spearman correlation coefficient r_s in TAVI/PAVI

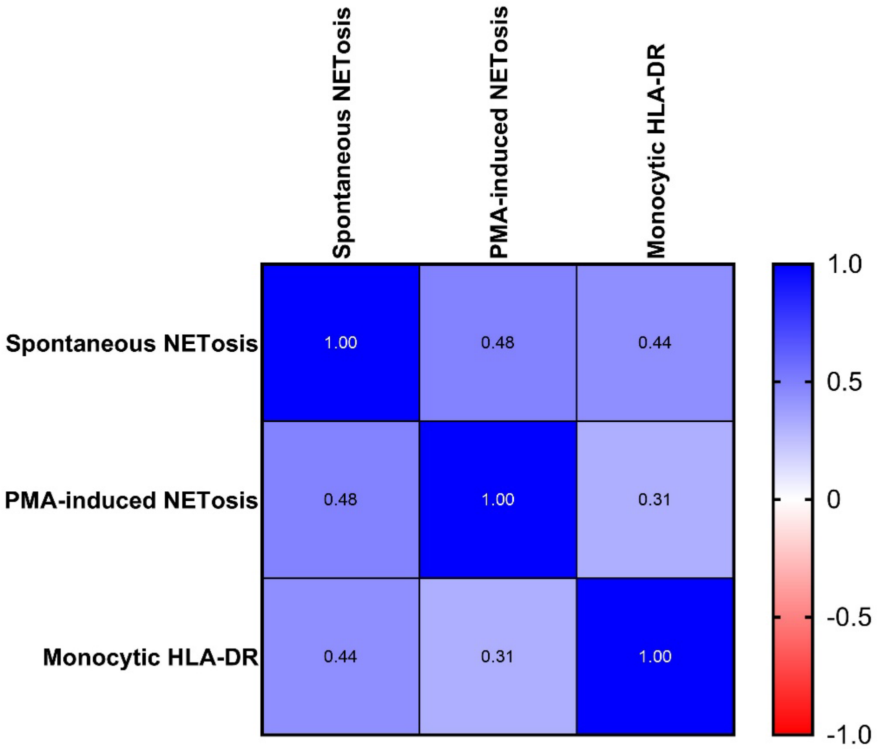

Spearman correlation coefficient r_s in ACB

Fig. S1 Correlation between monocytic HLA-DR and neutrophilic NETosis-related endpoints. Top: Heat map of Spearman's rank correlation coefficient values for each immune endpoint pairing in the TAVI/PAVI patient sample set. The monocytic HLA-DR showed a positive correlation trend with the spontaneous NETosis (p=0.063) and no correlation with the PMA-induced NETosis (p=0.968). Bottom: Heat map of Spearman's rank correlation coefficient values for each immune endpoint pairing in the ACB patient sample set. The monocytic HLA-DR was significantly correlated with the spontaneous NETosis (p=0.008) and showing a positive correlation trend with the PMA-induced NETosis (p=0.067). Between NETosis-endpoints, the PMA-induced NETosis was significantly correlated with the spontaneous NETosis in ACB sample set (p=0.003).

Supplemental figure 2

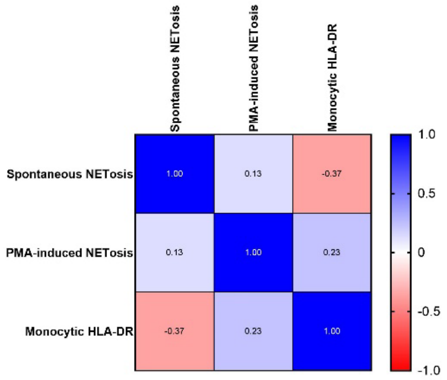

Spearman correlation coefficient r_s in TAVI - T1

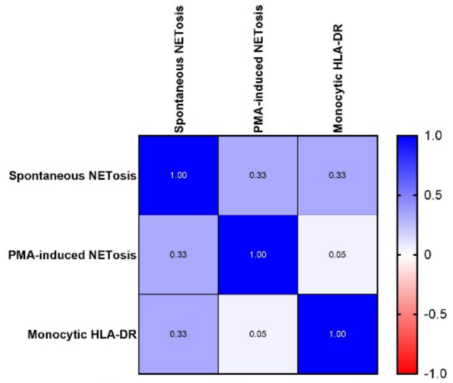

Spearman correlation coefficient r_s in ACB-T1

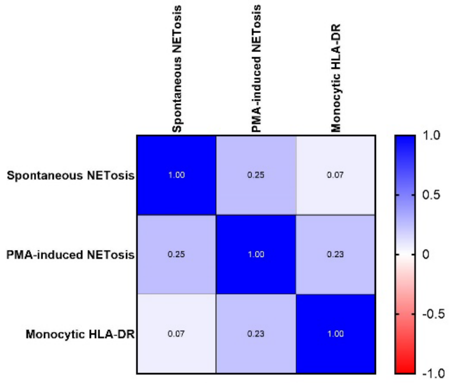

Spearman correlation coefficient r_s in TAVI - T2

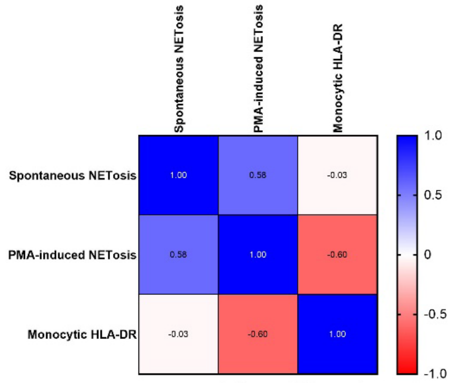

Spearman correlation coefficient r_s in ACB-T2

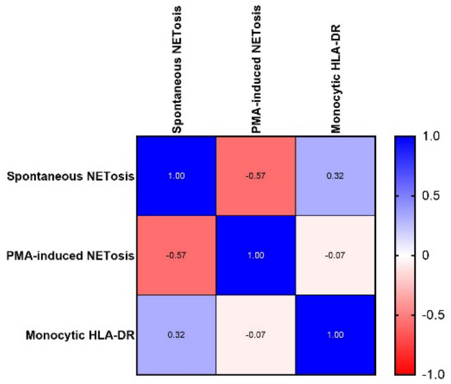

Spearman correlation coefficient r_s in TAVI - T3

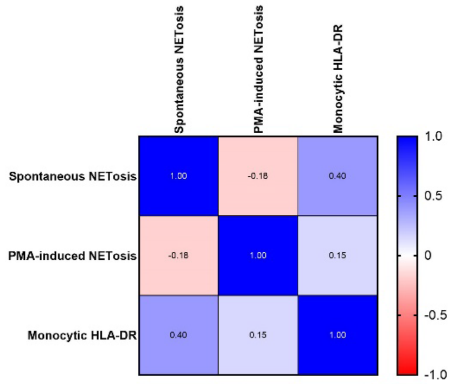

Spearman correlation coefficient r_s in ACB-T3

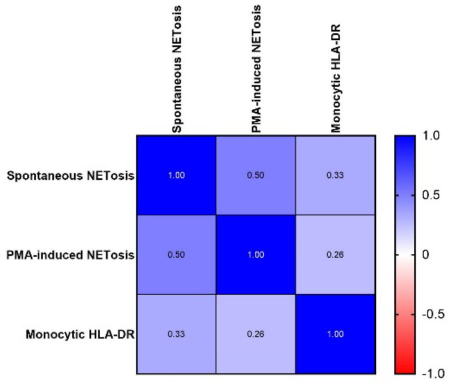

Spearman correlation coefficient r_s in TAVI - T4

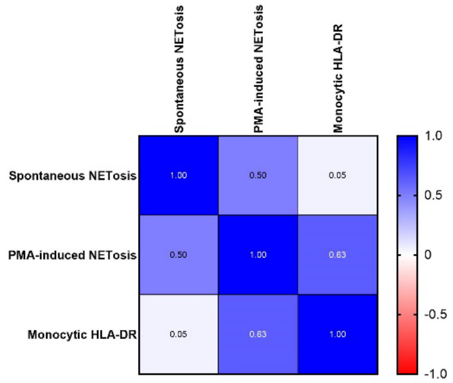

Spearman correlation coefficient r_s in ACB-T4

Fig. S2 Correlation between monocytic HLA-DR and neutrophilic NETosis endpoints at specific surgical timepoints. Left, heat maps of Spearman's rank correlation coefficient values for each immune endpoint pairing in the TAVI/PAVI patient sample set at single surgical time points T1-T4. Right, heat maps of Spearman's rank correlation coefficient values for each immune endpoint pairing in the ACB patient sample set at single surgical time points T1-T4.

T1: before surgery, T2: end of surgery, T3: 24h after surgery, and T4: 48h after surgery.

Supplemental figure 3

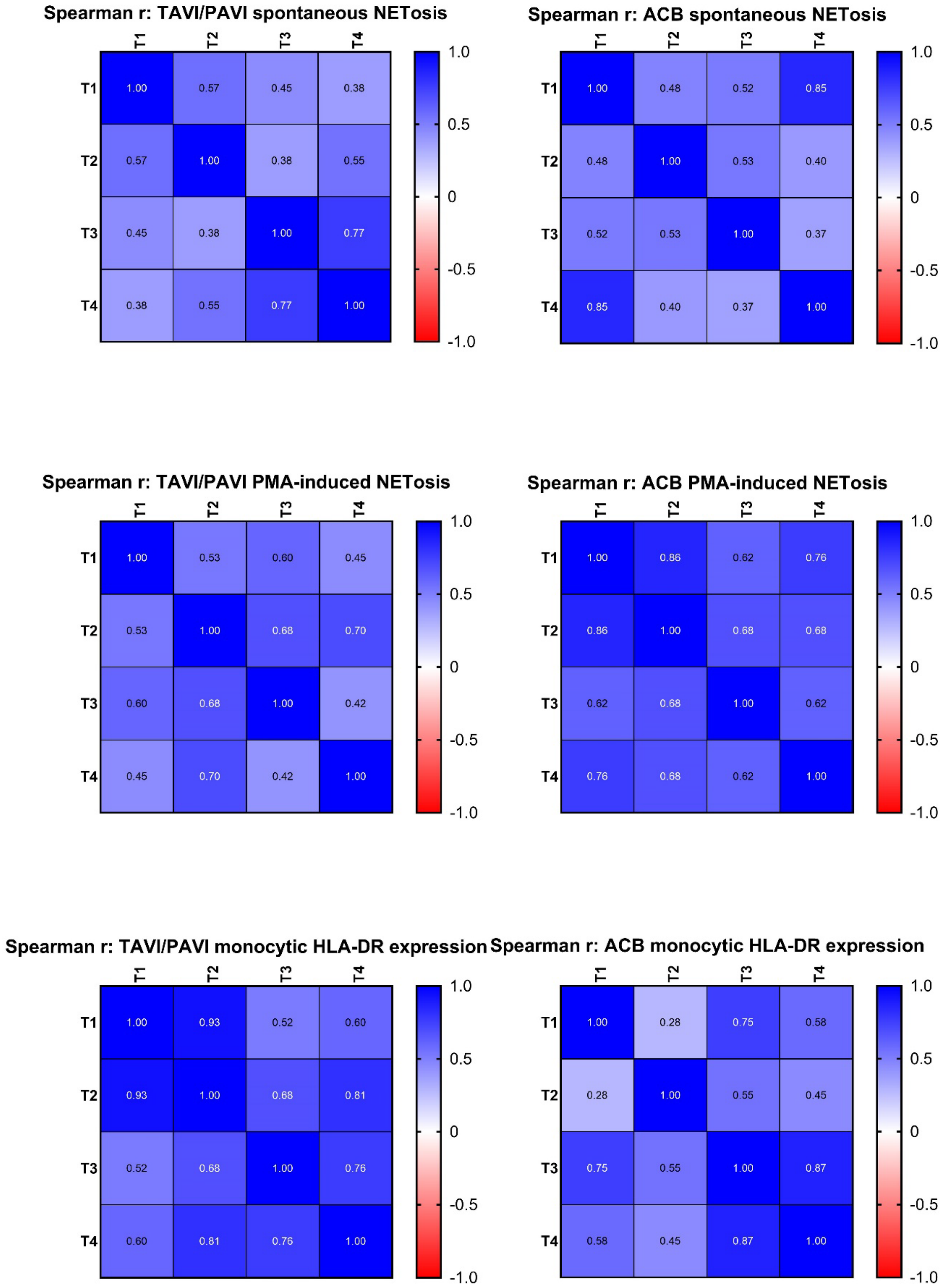

Fig. S3 Correlation between specific immune endpoints at different surgical timepoints. Left, heat maps of Spearman's rank correlation coefficient values of a given immune endpoint for each surgical timepoint pairing in the TAVI/PAVI patient sample set. Right, heat maps of Spearman's rank

correlation coefficient values of a given immune endpoint for each surgical timepoint pairing in the ACB patient sample set. In the TAVI/PAVI dataset, for all endpoints a general positive correlational trend was found across different surgical time. Significant correlations were found between T3 and T4 for the spontaneous NETosis (p=0.021); between T2-T3 (p=0.050) and T2-T4 (p=0.043) for the PMA-induced NETosis; and between T1-T2 (p=0.001), T2-T3 (p=0.050), T2-T4 (p=0.022), and T3-T4 (p=0.037) for monocytic HLA-DR expression. In the ACB dataset, Significant correlations were found between T1 and T4 for the spontaneous NETosis (p=0.006); between T1-T2 (p=0.011), T1-T4 (p=0.037), T2-T3 (p=0.050), and T2-T4 (p=0.050) for the PMA-induced NETosis; and between T1-T3 (p=0.015) and T3-T4 (p=0.002) for monocytic HLA-DR expression.

T1: before surgery, T2: end of surgery, T3: 24h after surgery, and T4: 48h after surgery.

Supplemental figure 4

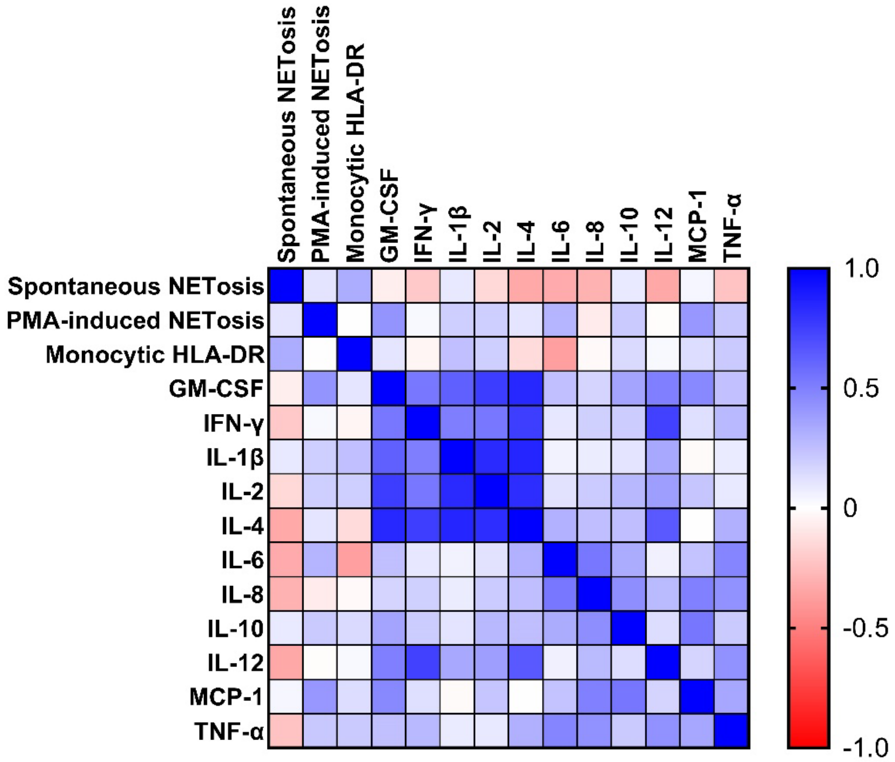

Spearman correlation coefficient r_s in TAVI/PAVI

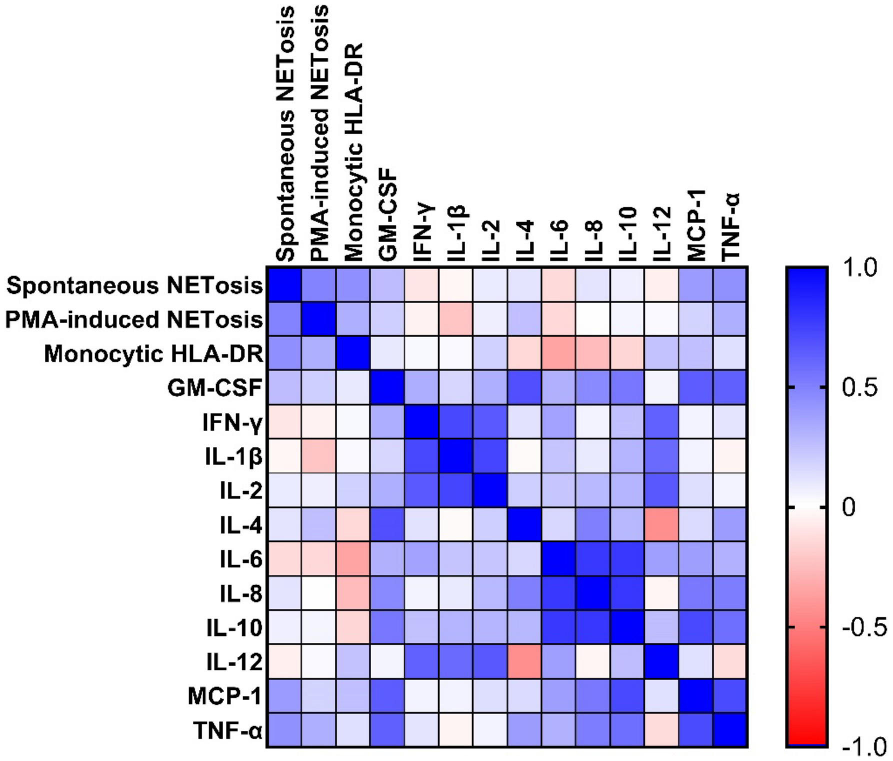

Spearman correlation coefficient r_s in ACB

Fig. S4 Correlation between inflammatory cytokines/chemokine and immunocyte-related endpoints. Top: Heat map of Spearman's rank correlation coefficient values for each immune endpoint pairing in the TAVI/PAVI patient sample set. Bottom: Heat map of Spearman's rank correlation coefficient values for each immune endpoint pairing in the ACB patient sample set. The nonparametric Spearman correlation coefficient, rs, and the corresponding two-tailed (two-sided) P values are listed in supplementary Table 1 and 2.
